# Community Health Worker Optimization of Antihypertensive Care in HIV (COACH): Study protocol for a pilot trial of an intervention to improve hypertension care among Tanzanians with HIV

**DOI:** 10.1371/journal.pone.0315027

**Published:** 2024-12-17

**Authors:** Wai Yan Min Htike, Preeti Manavalan, Lisa Wanda, Kelvin Haukila, Blandina T. Mmbaga, Francis M. Sakita, Rennyda Zebedayo, Francis Gwasma, Tazeen Jafar, Hayden B. Bosworth, Nathan M. Thielman, Julian T. Hertz

**Affiliations:** 1 Division of Natural and Applied Science, Duke Kunshan University, Kunshan, Jiangsu, China; 2 Duke Global Health Institute, Durham, NC, United States of America; 3 Department of Medicine, University of Florida College of Medicine, Gainesville, FL, United States of America; 4 Kilimanjaro Clinical Research Institute, Moshi, Tanzania; 5 Kilimanjaro Christian Medical University College, Moshi, Tanzanai; 6 Department of Emergency Medicine, Kilimanjaro Christian Medical Centre, Moshi, Tanzania; 7 Majengo Health Centre, Moshi, Tanzania; 8 Pasua Health Centre, Moshi, Tanzania; 9 Program in Health Services & Systems Research, Duke-NUS Medical School, Singapore, Singapore; 10 Department of Renal Medicine, Singapore General Hospital, Singapore, Singapore; 11 Duke Clinical Research Institute, Duke University, Durham, NC, United States of America; 12 Department of Population Health Sciences, School of Medicine, Duke University, Durham, NC, United States of America; 13 Division of Infectious Diseases, Department of Medicine, Duke University Medical Center, Durham, NC, United States of America; 14 Department of Emergency Medicine, Duke University Medical Center, Durham, NC, United States of America; Public Library of Science, UNITED KINGDOM OF GREAT BRITAIN AND NORTHERN IRELAND

## Abstract

**Objective:**

This study will evaluate the feasibility and preliminary effectiveness of the COACH (Community Health Worker Optimization of Antihypertensive Care in HIV) intervention, which integrates hypertension management into existing HIV care for people living with HIV (PLWH) in Tanzania.

**Methods:**

The study will be conducted at two HIV Care and Treatment Centers (CTCs) in Tanzania. In a single-arm pre-post feasibility trial, 100 PLWH with hypertension will be enrolled and will receive the six-month intervention. The COACH intervention includes six monthly hypertension educational sessions delivered by community health workers (CHWs) and integrated within HIV CTC visits, monthly blood pressure monitoring, follow up care coordination, fully subsidized antihypertensive medications, a standardized antihypertensive treatment algorithm, and training for providers. The intervention’s implementation outcomes will be evaluated using the Reach Effectiveness Adoption Implementation Maintenance (RE-AIM) framework, and the primary study outcome (reach of the intervention) will be the proportion of all scheduled intervention sessions attended by participants, a measure of feasibility. Secondary clinical effectiveness outcomes will include adherence to antihypertensive medication, blood pressure control, body mass index, cardiovascular risk, and hypertension knowledge.

**Significance:**

The COACH intervention has the potential to significantly improve hypertension management among PLWH in Tanzania by leveraging the existing HIV care infrastructure and CHWs. This study will provide crucial insights into the feasibility and potential effectiveness of the intervention in integrating hypertension care into HIV services, informing larger-scale implementation and policy changes in Tanzania and other resource-limitted settings.

**Trial registration:**

Clinical trials.gov Identifer: NCT06503991.

## Introduction

In sub-Saharan Africa (SSA), people living with HIV (PLWH) face an accelerating epidemic of noncommunicable diseases (NCDs), including hypertension [1, 2], which is a leading risk factor for cardiovascular disease (CVD) and death globally [3–5]. Multiple studies have demonstrated a large burden of uncontrolled hypertension among PLWH in SSA [6–12]. For instance, in a cohort of 500 PLWH with a mean age of 45.3 (SD, 11.4) years [13] in Tanzania, 35% had hypertension, the majority of whom were unaware of their hypertension diagnosis and were not taking antihypertensive medications [13]. Concerningly, 31% of hypertensive Tanzanians with HIV in this cohort had ischemic changes on electrocardiography and greater than half had intermediate to high 10-year risk for an atherosclerotic CVD event [14, 15]—indicating that these patients are on an accelerated pathway towards premature CVD-related morbidity and mortality. In SSA, a region which bears the highest burden of HIV globally, the combined impact of chronic HIV infection and hypertension markedly increases the risk for premature CVD as PLWH age [16–18]. As such, there is an urgent need for tailored interventions to mitigate the epidemic of preventable CVD-related morbidity and mortality in this population.

Tailored interventions must be developed in consideration of the cultural context and the barriers to the provision of hypertension care faced by PLWH in SSA [19]. For example, prior qualitative research in Tanzania identified multiple clinician shortages, fragmented primary healthcare systems, high costs of care, and a lack of training for both patients and clinicians in managing NCDs [2, 20–23]. Community health worker (CHW)-delivered care and integrated HIV and NCD care, are two models which, when combined, may effectively address these critical barriers to hypertension care in Tanzania and other similar settings. CHWs are existing, valuable, members of the HIV care team in SSA and routinely provide HIV testing, counseling and care coordination. However, CHWs do not routinely provide NCD care or counseling for PLWH [24]. Shifting tasks to CHWs has improved NCD control among general populations in resource-limited settings [25–27]. In Tanzania, where physicians may manage close to 50 patients per day [28] and have limited time for hypertension screening and management, task-shifting hypertension care to CHWs holds considerable promise [21, 29]. In addition, integration of HIV and NCD care can help address the challenges of fragmented and siloed health systems [30]. Specifically, by utilizing the robust existing HIV care infrastructure, which effectively supports long-term HIV care retention, HIV clinics in Tanzania (Care and Treatment Centers) are well-positioned to engage PLWH in managing other chronic conditions, such as hypertension [31, 32].

Guided by the ADAPT-ITT framework [33], our team used community-based participatory design principles [34–36] to iteratively develop the COACH (CHW Optimization of Antihypertensive Care in HIV) intervention. COACH adapts elements from the evidence-based **Control of Blood Pressure and Risk Attenuation (COBRA)** intervention, demonstrated to be effective in Southeast Asia, and the **CHW-delivered Hypertension Management Pilot (CHAMP)**​ intervention, which was developed and piloted in Tanzania [24, 37–41]. A team of key stakeholders, including Tanzanian HIV and hypertension clinicians, CHWs, community members and patients with HIV and hypertension, nurses, pharmacists, healthcare administrators, and policymakers, adapted the evidence-based COBRA intervention by incorporating key elements of CHAMP to develop a tailored, multicomponent intervention aimed to improve hypertension care among Tanzanians with HIV. While COBRA has been proven effective in general community populations in Asia, its adaptation to integrate CHAMP elements uniquely addresses barriers to hypertension care among PLWH in SSA [37–40, 42–45]. Systematic use of the ADAPT-ITT framework and community-based participatory design for intervention adaption ensured that those who would benefit from the intervention drove its creation, thus meeting local needs for hypertension care. The COACH intervention includes CHW-delivered hypertension health education, blood pressure monitoring, provider training and treatment algorithms​, antihypertensive medication subsidies, and follow-up tracking all integrated within the HIV clinical setting.

This pilot trial will determine key implementation outcomes of COACH, guided by the Reach Effectiveness Adoption Implementation Maintenance (RE-AIM) framework [46–48], such as reach (i.e., feasibility), adoption, and fidelity, and will also generate effect size estimates of clinical outcomes of the intervention to address hypertension among adults living with HIV in Tanzania. The results of this trial will have important implications for policymakers, clinicians, and PLWH across SSA and the methods described in this protocol paper could be adapted by others testing similar interventions for hypertension care among PLWH in resource-limited settings.

## Materials and methods

### Study setting

Participants of our study will be recruited from two HIV Care and Treatment Centers (CTCs) in northern Tanzania: Majengo Care and Treatment Center (MCTC) and Pasua Care and Treatment Center (PCTC). These clinics are located in a region with a high community prevalence of hypertension (28% in 2014 among the population with mean age of 45.0 years (IQR 35–59)) [49], a high community prevalence of HIV (4.0% in 2023) [50], and substantial undertreatment of hypertension among PLWH [6, 8]. MCTC and PCTC provide comprehensive HIV care to over 2300 adults, and both are government-funded HIV clinics offering free HIV care, including free antiretroviral therapy (ART). However, these clinics do not consistently offer hypertension screening or care [20].

### Study design

**Fig 1:**
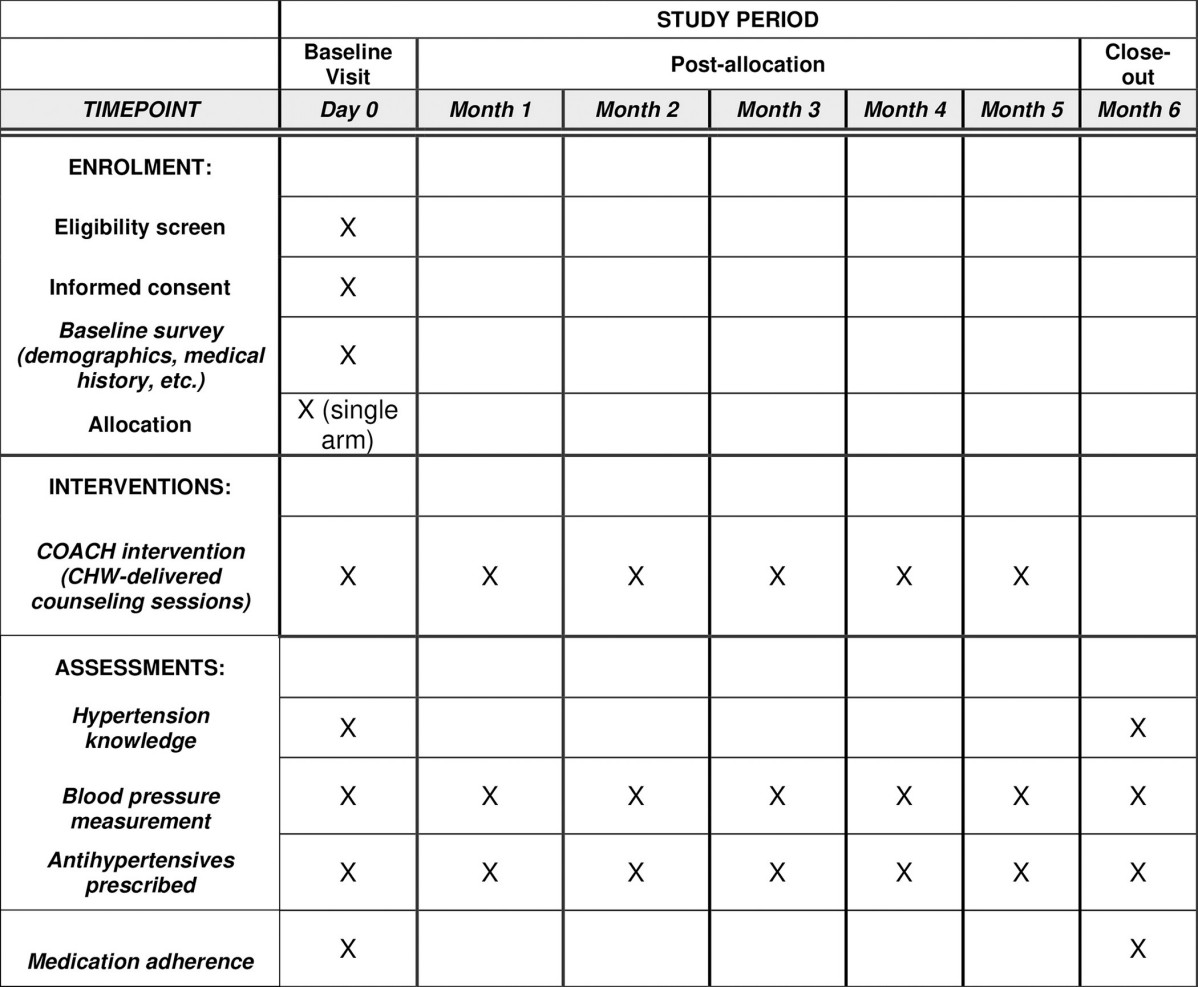
SPIRIT diagram of study procedures, interventions, and assessments.

This single-arm pilot trial aims to evaluate the reach, adoption, fidelity, and preliminary effectiveness of the COACH intervention for improving hypertension care among PLWH in Tanzania. To understand the implementation context and facilitate the ability to scale the adapted intervention (COACH), we will employ the RE-AIM framework [46–48]. Both quantitative and qualitative measures will be used to assess implementation outcomes; secondary clinical effectiveness outcomes will be assessed via comparison of blood pressure control and medication adherence at baseline and at the conclusion of the six-month intervention. The overall schedule of study procedures, interventions, and assessments are presented in Fig 1.

### The COACH intervention

**Fig 2:**
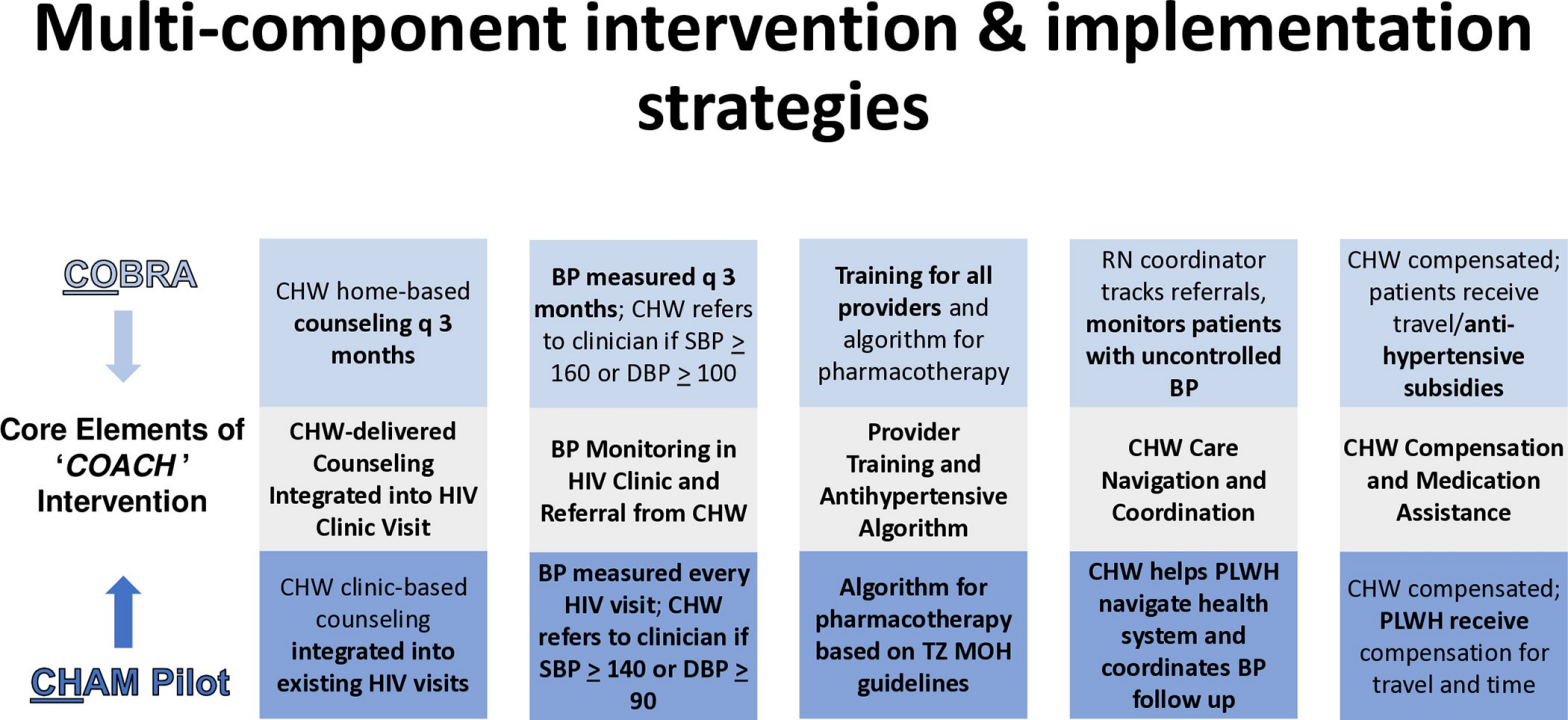
Overlap of core components of COBRA and CHAMP which will be integrated and adapted in COACH.

The COACH intervention is an adapted, multi-component strategy designed to improve blood pressure control among PLWH in Tanzania. The intervention integrates key elements from two interventions, the evidence-based COBRA intervention and the CHAMP intervention [24, 37–41], to address the unique healthcare needs of hypertensive PLWH within the HIV clinic setting. The overlapping core components of COACH from COBRA and CHAMP can be seen in Fig 2.

The COACH intervention was adapted from core elements of COBRA and CHAMP using the ADAPT-ITT model [33] and community based-participatory design [34–36] to guide and inform the adaptation process. Using previously collected data to identify barriers and facilitators of hypertension care for PLWH in Tanzania [2, 20–23], an Intervention Design Team (IDT) tailored the COBRA structure to the needs of PLWH in Tanzania considering (a) data generated from CHAMP, (b) current guidelines and policies, and (c) existing clinic infrastructure [24, 37]. The IDT (key stakeholders including Tanzanian HIV and hypertension clinicians, nurses, pharmacists, CHWs, community members and patients with HIV and hypertension, healthcare adminstrators and policy makers) conducted a total of 14 biweekly workshops in Swahili on the original COBRA and CHAMP materials from published protocols, and through collaborative discussions and consensus-building with the study investigators, adapted and refined these interventions into the COACH intervention. “Theater testing” of COACH materials was conducted in order to refine intervention content [33]. The process of developing and refining COACH occurred over a seven-month period in 2024; the process of developing the COACH intervention will be published separately. The adapted COACH intervention included five key components, with details outlined in Table 1.

**Table 1:** Components of the COACH intervention for integrating hypertension and HIV care in Tanzania.

Component	Details
1. CHW Counseling	• Six one-on-one counseling sessions between clinic-based CHW and patient, on a monthly basis • Each session lasts for approximately 30 minutes and is guided by a curriculum with hypertension educational content • Brief, telephone check-in sessions between each in-person session focussed primarily on antihypertensive medication adherence • Patient receives educational handout
2. Monitoring and Referrals	• CHW measures patient’s blood pressure on arrival to the clinic at each monthly visit • CHW refers patient to provider in the CTC for initiation or modification of antihypertensive therapy if BP is elevated (systolic ≥ 140 mmHg or diastolic ≥ 90 mmHg) • Referrals for antihypertensive care are made to patient’s regular HIV provider, rather than to a separate provider
3. Provider Training and Antihypertensive Algorithm	• All HIV providers receive 2 two-hour training sessions led by a hypertension specialist and a refresher training in 3 months. • Training sessions cover hypertension diagnosis and management, as well as common treatment misconceptions • Providers follow a standardized hypertension treatment algorithm based on WHO guidelines, with stepwise instructions for titration of therapy
4. CHW Care Navigation and Coordination	• CHWs maintain a registry of patients with HIV and hypertension • CHWs monitor patient attendance at monthly appointments and contact patients who miss appointments via telephone to re-schedule their visit • Antihypertensives dispensed directly from the HIV clinic pharmacy, rather than from a separate pharmacy • Patients with HIV and hypertension are registered in the general clinic electronic medical record system to allow for integration of their hypertension care into existing medical records system
5. Subsidies	• Antihypertensives in the treatment algorithm (amlodipine, losartan, and combined losartan/hydrochlorothiazide) provided for free to participants • CHWs receive a monthly supplemental stipend for providing hypertension counseling and care coordination

Briefly, the COACH intervention will be delivered over six months in each CTC and will incorporate CHW-delivered hypertension counseling and blood pressure monitoring into existing HIV clinic appointments. Persistently elevated blood pressure will trigger a referral to a prescribing provider within the HIV clinic. CHWs will coordinate and track these referrals, ensuring follow-up and management. Participants will meet in-person monthly with the CHW in the CTC to receive a total of six CHW-delivered educational counselings sessions. These sessions will cover counseling for hypertension education, medication adherence, lifestyle modifications, and regular blood pressure monitoring. The first session will occur on the day of enrollment. If blood pressure remains above goal, the CHW will directly refer the participant to the CTC clinician for pharmacologic antihypertension treatment in accordance with the COACH treatment algorithm and WHO guidelines. CTC providers will receive training on hypertension management and use of the COACH treatment algorithm prior to start of the intervention. Participants will receive antihypertensive medications that are included in the COACH treatment algorithm at no cost for the duration of the study. The CHW will call participants in-between each in-person session for a brief check in, to remind about future appointments and to briefly discuss any challengs with the prescribed antihypertensive agents.

### Study participants (inclusion and exclusion criteria)

#### Inclusion criteria

Participants must be at least 18 years old and living with HIV, currently enrolled in care at either MCTC or PCTC. Trained research assistants will measure the blood pressure of all patients presenting to MCTC or PCTC and offer them inclusion in the study if they meet eligibility criteria. Only patients meeting the study definition of hypertension will be eligible for inclusion. Hypertension will be defined as follows: Participants with a single systolic blood pressure (SBP) measurement greater or equal to 160 mmHg and/or diastolic blood pressure (DBP) greater or equal to 100 mmHg will be invited to participate. Additionally, participants with an SBP between 140–159 mmHg and/or DBP between 90–99 mmHg and prior documented elevated blood pressure (SBP greater or equal to 140 mmHg and/or DBP greater or equal to 90 mmHg) will also be invited to participate. Finally, participants with an SBP between 140–159 mmHg and/or DBP between 90–99 mmHg and no prior documented elevated blood pressure will be asked to return to the clinic within 1 to 2 weeks for a repeat measurement; those with two separate measurements of SBP greater than or equal to 140 mmHg and/or DBP greater than or equal to 90 mmHg will also be eligible for participation.

#### Exclusion criteria

Pregnant women will be excluded due to the potential risks and complications associated with hypertension management during pregnancy and as pregnant women with HIV in Tanzania currently receive HIV care and services at a separate antenatal clinic rather than the CTC. Individuals who do not provide informed consent will not be eligible to participate in the study.

#### Sample size

As this is a pilot feasibility trial using a pre-post analysis, our focus will be on assessing implementation outcomes. The primary outcomes of this study are implementation outcomes (see *Study outcomes* below), and no prior assumptions were made about effect size for secondary clinical effectiveness outcomes and the pilot trial will not be powered to detect significant intervention effects or stable effect sizes. Therefore, we will enroll 100 participants (50 from MCTC and 50 from PCTC) to assess reach, adoption, and fidelity of the COACH intervention. We will also gather preliminary clinical effectiveness data to generate parameter estimates and ranges of values and calculate eligibility, recruitment and attrition rates to inform sample size calculations, power considerations and potential effects for the design of a larger, definitive, fully powered randomized trial in the future.

#### Study procedures

During regular HIV care appointments, research assistants will screen patients while they are in the clinic waiting areas to identify individuals meeting the study definition for hypertension. Patients identified with hypertension will be informed about the research study. If interested in participating, they will be escorted to a private area within the clinic to complete the written informed consent and the baseline survey.

The baseline survey will assess demographics, medical history, hypertension knowledge (via the Hypertension Knowledge Level Scale [51]), and antihypertensive medication use and adherence. Data collection at baseline will include blood pressure measurements, and weight, height, and waist circumference, measured directly by research assistants. Following the baseline survey all enrolled participants will receive the COACH intervention. Blood pressure measurements and antihypertensive medication prescription data will be collected during each of the monthly COACH intervention sessions.

Follow-up assessments will occur six months post-enrollment, using researcher-administered surveys and medical record reviews to evaluate blood pressure control, medication adherence, and other outcomes including BMI, hypertension knowledge, and NHANES CVD risk score (as described below).

#### In-depth interviews

Exit In-depth interviews (IDIs) will be conducted to collect qualitative data on participants’ experiences and perceptions of the COACH intervention. IDIs will be conducted with key stakeholders (patients, providers, CHWs, clinic administrators) until thematic saturation is achieved. We anticipate completing approximately 18 IDIs (approximately 6 patients, 6 providers, 2 CHWs, and 4 administrators), using purposive sampling to recruit participants from a diversity of backgrounds. These IDIs will be audio-recorded, and transcribed verbatim for data analysis. Informed consent will be obtained from all IDI participants prior to participation, and all IDI participants will receive a reimbursement of 5,000 Tanzanian shillings (approximately 2 USD) for their time and travel.

#### Data management

All research data will be collected on encrypted tablets using the Open Data Kit (ODK) platform, uploaded directly to a secure encrypted server at the KCMC-Duke Collaboration Data Room, and then be transferred and stored on secure servers at Duke University. IDIs and intervention sessions will be audio recorded, translated and transcribed with any identifying information removed during transcription. Transcriptions will be saved to a Duke secure server, at which time the audio files will be permanently deleted. Data sets will be downloaded for storage on a secure HIPAA-compliant password-protected server (Duke Box) managed by Duke University.

No results will be reported in a personally identifiable manner.

Furthermore, data monitoring will take place to ensure the integrity and accuracy of the information collected. All study protocols will be implemented and conducted according to guidelines for good clinical practice (GCP). All study personnel have completed training in GCP.

### Safety considerations

Informed consent will be obtained in a private setting to maintain confidentiality and ensure that participants understand the information provided. Any adverse events encountered during the study will be promptly documented, addressed, and reported to the Institutional Review Board (IRB) committees at KCMC, Tanzania National Institute for Medical Research (NIMR), and Duke Health.

Participants with hypertension will receive regular monitoring throughout the study. In cases of concern for hypertension emergency, an immediate referral to a prescribing provider within the clinic will be initiated. Regular monitoring and follow-up will ensure that participants receive appropriate care. CHWs will play a key role in coordinating and tracking referrals, and ensuring that participants with severe hypertension or other health issues receive the necessary follow-up care. Through these comprehensive safety measures, the study aims to safeguard the well-being of all participants while achieving its research objectives.

### Study outcomes

This study will focus on implementation outcomes of the COACH intervention, guided by the RE-AIM framework [47, 48]. Secondary preliminary effectiveness outcomes will also be evaluated to inform future large-scale trials of COACH.

### Primary outcome

The primary outcome of this pilot trial will be the reach (i.e., feasibility) of the COACH intervention, which will primarily be measured by proportion of total scheduled intervention sessions attended by study participants over the course of the 6-month intervention period. This outcome is designed to measure the feasibility of the COACH intervention in a real-world setting.

### Secondary implementation outcomes

The evaluation of secondary implementation outcomes will focus on four key aspects: reach, adoption, implementation, and maintenance.

*Reach*. Reach evaluates the extent of participation and feasibility of the intervention (i.e., the ability of the intervention to ‘reach’ the intended population). In addition to our primary reach/feasibility outcome, we will use counts and proportions to describe: 1) the proportion of eligible participants who are recruited and enrolled in the pilot trial, 2) the proportion of participants attending referral appointments with a doctor, 3) the proportion of participants prescribed antihypertensives, and 4) the proportion of prescribed antihypertensives conforming to the COACH treatment algorithm.

*Adoption*. Adoption assesses whether the intervention can be incorporated into routine practice and its acceptability to participants and providers. We will conduct exit IDIs with a subset of COACH participants (approximately n = 6), and with physicians, nurses, CHWs, and administrators from MCTC and PCTC (approximately n = 12). These interviews will explore barriers and facilitators to intervention adoption, perceived benefits, favorite and least favorite aspects of the intervention, and suggestions for modification and scale-up. Additionally, the interviews will investigate reasons for non-participation or incomplete participation in COACH sessions. We will incorporate the four items of the Acceptability of Intervention Measure (AIM) [52] and use the Theoretical Framework of Acceptability (TFA) to code responses based on seven key domains: affective attitude, burden, perceived effectiveness, ethicality, intervention coherence, opportunity costs, and self-efficacy [53], as described below (see *Data analysis*).

*Implementation*. Implementation examines the fidelity to the intervention content. To ensure adherence to the COACH content, we will audio record and review approximately 10% of CHW-delivered counseling sessions. These recordings will be assessed for session completion and the use of core counseling components [54]. CHWs will also complete a COACH fidelity checklist for each session. Counts and proportions will be used to report fidelity metrics.

*Maintenance*. Maintenance measures the sustainability of the intervention. We will conduct IDIs with providers (approximate n = 6), administrators (approximate n = 2), CHWs (approximate n = 2) from MCTC and PCTC, and representatives from the Ministry of Health (MOH) (approximate n = 2). These discussions will evaluate the resources, strategies, and policies necessary for sustaining the COACH intervention. Participants will be asked about their willingness to continue participating in COACH and their perspectives on integrating COACH into routine care in Tanzania.

### Secondary effectiveness outcomes

Secondary effectiveness outcomes will focus on patient-oriented indicators relevant to hypertension management. These outcomes include:

- Adherence to antihypertensive medication: The proportion of participants reporting adherence to antihypertensive medication at the 6-month follow-up. Medication adherence will be assessed through the Voils measure [55].
- Blood Pressure Control: The proportion of participants with controlled blood pressure, defined as SBP less than 140 mmHg and DBP less than 90 mmHg, at 6-month follow-up. Blood pressure measurements at baseline and at 6-month follow up will be measured directly by research assistants.
- Hypertension Knowledge: Changes in participants’ knowledge about hypertension will be evaluated by comparing participants’ scores on the Hypertension Knowledge-Level Scale at baseline and at the 6-month follow-up [51].
- BMI: Changes in BMI from baseline to the 6-month follow-up will be recorded to assess the impact of the intervention on weight management. BMI will be calculated directly from weight and height measurements taken during clinic visits.
- NHANES CVD risk score: Changes in the NHANES CVD risk score [56, 57] from baseline to the 6-month follow-up will be assessed. This score, derived from the National Health and Nutrition Examination Survey, incorporates various health metrics to estimate the risk of developing CVD. The NHANES CVD risk score was chosen over other scores because it does not require laboratory testing, and it performs as well as other risk scores in predicting cardiovascular events [56, 57].

### Data analysis

Quantitative analysis will be performed in the R Suite. Descriptive statistics will be employed to summarize the baseline characteristics of the study participants. Measures of central tendency (mean, median) and dispersion (standard deviation, interquartile range) will be used for continuous variables, while frequencies and percentages will be used for categorical variables. Paired t-tests will be used to compare differences in continuous variables (such as participant blood pressure readings) at baseline and at six-month follow-up. The McNemar test will be used to compare differences in categorical variables (such as medication adherence) at baseline and at six-month follow-up.

A rapid qualitative analysis approach will be used for the analysis of IDI transcripts [58]. This is a pragmatic and rigorous method used to obtain actionable qualitative data on a shorter timeline and facilitates the collection of readily applied qualitative data to inform processes of implementation. Qualitative analysis will be informed by the Theoretical Framework of Acceptability (TFA)​ [53]. Transcripts will be independently coded by at least two members of the coding team, and discrepancies in codes will be resolved by discussion and consensus-building.

### Study timeline

- Training (Q4 2024): Training of CHWs and other relevant staff on the COACH intervention and study procedures.
- Pilot Enrollment (Q1 2025—Q4 2025): Enrollment of participants into the pilot study, implementation of the COACH intervention, and follow-up visits. Recruitment will begin in January 2025 and conclude by December 2026.
- IDIs (Q3 2025—Q4 2025): Conducting IDIs to gather qualitative data on the feasibility and acceptability of the intervention.
- Data Analysis (Q3 2025 –Q1 2026): Analysis of collected data to evaluate primary and secondary outcomes and prepare findings for dissemination.
- Study timeline is presented in Table 2.

**Table 2:** Study timeline.

	2024				2025				2026
Quarter	Q1	Q2	Q3	Q4	Q1	Q2	Q3	Q4	Q1
Planning & Training									
Pilot Enrollment (Aim 2)									
IDIs									
Data analysis									

### Ethical approval

This study has been reviewed and approved by IRBs at Duke Health (PRO00090902 approved on July 22^nd^, 2024), NIMR (NIMR/HQ/R.8a/Vol.IX/4615, approved on May 21^st^, 2024), and KCMC (Proposal 1454, approved on February 12^th^, 2024). All participants will provide written informed consent prior to enrollment.

## Discussion

This manuscript presents the study protocol for a pilot trial of an intervention, uniquely derived from COBRA and CHAMP with broad community-driven input, to improve hypertension management among PLWH in Tanzania. We anticipate that the COACH intervention will be highly feasible and acceptable and enhance the management of hypertension among PLWH in Tanzania, resulting in improved blood pressure control and increased knowledge about hypertension. By integrating hypertension management into existing HIV care and utilizing task-shifting through CHW-delivered interventions, we expect to demonstrate feasibility of this approach in a real-world setting. We anticipate that the results of this study will inform future changes in healthcare policy and practice in Tanzania, potentially leading to the adoption of integrated care models that address both HIV and NCDs. This could ultimately improve the health outcomes and quality of life for PLWH and contribute to the broader global health goal of managing NCDs in low-resource settings [2].

This study will have several limitations that should be acknowledged. First, as a pilot feasibility trial, the sample size is relatively small, which will limit our ability to precisely estimate effect sizes. Secondly, the study’s duration is limited to only six months; additional studies will be needed to assess long-term adherence and health outcomes following implementation of COACH. Another limitation of this study is the reliance on self-reported data for some measures, such as medication adherence, which may be subject to recall bias or social desirability bias. Finally, the study will be conducted in two urban CTCs in Tanzania, which may limit the generalizability of our findings to other regions and patient populations.

In conclusion, this study will determine the feasibility and preliminary effectiveness of the COACH intervention for utilizing a task-shifting approach and integrating hypertension care into HIV treatment services in Tanzania. The expected outcomes and impacts of this intervention could have significant implications for healthcare delivery in similar low-resource settings. Future multisite trials will be needed to determine the effectiveness of the intervention in a larger population and assess long-term impacts on health and cardiovascular events. The next steps will involve analyzing the data collected during this pilot trial, disseminating the results through peer-reviewed publications and conferences, and preparing for a fully powered trial to evaluate the intervention’s scalability and long-term effectiveness across multiple sites in Tanzania.

## Supporting information

S1 Checklist SPIRIT 2013 checklist: Recommended items to address in a clinical trial protocol and related documents*. (DOCX)

S1 File (DOCX)
