# Supplementary material for: Community Health Worker Optimization of Antihypertensive Care in HIV (COACH): Study protocol for a pilot trial of an intervention to improve hypertension care among Tanzanians with HIV
Source: PLoS One. 2024 Dec 17;19(12):e0315027. doi: 10.1371/journal.pone.0315027 (PMC11651563; doi:10.1371/journal.pone.0315027)
Supplement: S1 File — (DOCX) [file pone.0315027.s002.docx]

**A COMMUNITY HEALTH WORKER-DELIVERED PROGRAM TO IMPROVE HYPERTENSION CARE AMONG TANZANIANS WITH HIV**

**Principal Investigator**

Francis Sakita, MD

Emergency Medicine

Kilimanjaro Christian Medical Centre

**Co-Investigators:**

Blandina T. Mmbaga, MD, PhD

Director, KCMC/Duke Collaboration

Kilimanjaro Christian Medical Center

Julian T. Hertz, MD

Associate Professor

Division of Emergency Medicine

Duke Global Health Institute

Preeti Manavalan, MD

Internal Medicine
University of Florida College of Medicine

Nathan Thielman, MD, MPH

Professor of Medicine and Global Heatlh
Duke University School of Medicine

Table of Contents

ABSTRACT: 3

Literature Review: 5

Rationale: 8

Broad Objective: 8

**Inclusion/ Exclusion Criteria:** 9

**Limitations of the Study:** 18

Budget Justification: 19

**Abstract:**

Background:

In sub-Saharan Africa (SSA), people living with HIV (PLWH) face a new epidemic of uncontrolled hypertension, the leading risk factor for death worldwide. Current care models in SSA are inadequate to address the growing burden of hypertension in PLWH, yet few interventions targeting this population exist. The Control of Blood Pressure and Risk Attenuation (COBRA) program, a multi-component program consisting of community-based CHW-delivered education and blood pressure monitoring and protocolized referrals to physicians, demonstrated efficacy in improving hypertension control and reducing all-cause mortality in a cluster-randomized trial across 3 countries in Asia. Evidence-based approaches like COBRA hold considerable promise in achieving hypertension control among PLWH in SSA but have not been studied in this population.

Objective:

In this study, we will adapt an evidence-based, multi-component, CHW-delivered hypertension intervention (COBRA) to improve hypertension care and reduce blood pressure among PLWH in Moshi, Tanzania.

Methods:

Using implementation science methods, we will adapt COBRA for delivery within the Tanzanian HIV clinic, examine implementation outcomes, and estimate effect size in preparation for a subsequent large-scale, hybrid effectiveness-implementation trial across Tanzania. We will assemble a Design Consultation Team of key stakeholders to assist in intervention adaption. Adaptation and of COBRA will occur iteratively over 8 months, guided by the ADAPT-ITT model (Assessment, Decision, Adaptation, Production, Topical Experts, Integration, Training, Testing), and informed by our previously collected qualitative data describing barriers and facilitators to hypertension care for PLWH. We will pilot the adapted intervention with a single arm pre-post feasibility trial in two HIV clinics and assess implementation (reach, adoptability, implementation, maintenance) and preliminary effectiveness outcomes (hypertension control, systolic and diastolic blood pressure, antihypertensive adherence, cardiovascular disease risk score, and hypertension knowledge). Our research team, comprised of experts in hypertension, HIV, and implementation science and our robust relationships with community agencies and health systems make us well-suited to conduct this work.

Intended Use of Results:

We anticipate that this study will produce one of the first locally-tailored interventions to improve hypertension care among Tanzanians with HIV. The results of this study will be used to inform future larger scalability and effectiveness studies.

**Abbreviations:**

ACS Acute Coronary Syndrome

CHW Community Health Worker

COBRA Control of Blood Pressure and Risk Attenuation

COBRA-TZ Adapted version of COBRA program for Tanzania

HIC High Income Countries

HIV Human Immunodeficiency Virus

KCMC Kilimanjaro Christian Medical Center

LMIC Low and Middle Income Countries

MCTC Majengo Care and Treatment Center

MI Myocardial infarction

PCTC Pasua Care and Treatment Center

PLWH People Living With HIV

SSA sub-Saharan Africa

WHO World Health Organization

**Introduction:**

Globally hypertension affects one billion people and leads to nine million deaths every year.^1^ Over 75% of these deaths are in low-middle income countries (LMIC).^1^ Sub-Saharan Africa has an estimated hypertension rate of 30-40% and poor compliance with follow-up.^2^ The prevalence of hypertension in the Moshi area of Tanzania is similar at 28%. ^3^ Hypertension is also often asymptomatic, which makes screening and compliance with treatment challenging.^4^

Globally, persons living with HIV (PLWH) have approximately double the risk of atherosclerotic cardiovascular disease compared to those without HIV.^5^ This heightened risk is thought to be due to vascular inflammation and increased predisposition to dyslipidemia and insulin resistance.^6,7^ Given the association between HIV and cardiovascular disease as well as improvements in HIV care, cardiovascular diseases are now the leading cause of death.^8^ The majority of PLWH live in sub-Saharan Africa (SSA), where the large burden of HIV co-exists with a rapidly increasing burden of traditional cardiovascular risk factors such as hypertension and obesity.^9-11^ Hypertension is the greatest attributable risk factor for cardiovascular disease in SSA, and places PLWH at additional risk for premature frailty, disability, and mortality.^12-14^ Despite the large burden of both HIV and cardiovascular risk factors in SSA, there has been very little study of hypertension and HIV in the region.^15,16^

This study will adapt and test the feasilbity of the Control of Blood Pressure and Risk Attenuation (COBRA), previously shown to be effective in a cluster randomized trial in Asia,^17-22^ for controlling hypertension among PLWH in SSA.

# **Literature Review:**

**Hypertension**

Globally hypertension disproportionately affects low-middle income countries. The World Health Organization describes this as a major public health problem, which accounts for 4.5% of the global disease burden and has lead to 64 million lost in disability- adjusted life years.^23^ The complications of hypertension are also preventable with affordable antihypertensive treatment. The treatment of hypertension has been estimated to prevent 40% of strokes and 15% of heart attacks.^23^ Treatment of even mild to moderate hypertension has been shown to be cost-effective and inclusion of an intervention program in addition to medication has lead to an Incremental cost-effectiveness ratio^24^ of 1,124 International Dollars per life-year gained in other developing countries.^25^

**Hypertension Screening**

Community based approaches and home screening methods have been conducted in Sub-Saharan Africa and Tanzania specifically. The community-based approach captured more hypertensive patients; however, follow-up for both groups was similarly poor (31%).^2^ A study conducted in resource-limited settings in Asia has shown that the multi-component counseling program Control of Blood Pressure and Risk Attenuation (COBRA) addressing patient education, provider training, coordination of care, and subsidizing care costs improved blood pressure control and functional independence.^17-22^ Importantly, COBRA has only been evaluated in the community setting in certain south Asian countries (Sri Lanka, Pakistan, Bangladesh); it is unknown (1) whether COBRA would be acceptable or effective in SSA where contextual factors are different, (2) whether COBRA would be effective or feasible in a clinic-based setting, (3) whether COBRA would be acceptable or effective in the PLWH population, and (4) how COBRA and its components would need to be adapted for the Tanzanian context. Given the urgent need for interventions to improve hypertension control among PLWH in SSA, we seek to answer these questions and determine if COBRA can be effectively adapted to the Tanzanian context. Although COBRA has not previously been evaluated in PLWH or in SSA, community data suggests that this program could be effective among PLWH in Moshi, Tanzania where over 90% of PLWH reported a desire for more education, were adherent to their HIV treatments, and expressed a willingness to change diet and lifestyle to improve cardiovascular health. At Majengo Care and Treatment Center (MCTC) in Moshi, Tanzania, prior research finds a 43% prevalence of hypertension among older (age ≥40 years) PLWH engaged with HIV-related care, indicating that this particular sub-population could greatly benefit from hypertension control programs.

Preliminary Data from Moshi

Similar to other studies in SSA, in the Kilimanjaro Region of Tanzania, we found high rates of hypertension among PLWH, but very low rates of blood pressure control. Among 500 PLWH, 35% had hypertension, 67% were unaware of their hypertension diagnosis, less than 3% were taking antihypertensive medications and 90% had uncontrolled blood pressure.^14,26^ Concerningly, 31% of hypertensive Tanzanians with HIV had ischemic changes on electrocardiography and greater than half had intermediate to high 10-year risk for an atherosclerotic CVD event^27,28^—indicating that these patients are on an accelerated pathway towards premature CVD-related morbidity and mortality.

**Statement of the Problem:**

Currently, PLWH in Sub Saharan Africa have a high prevalence of hypertension with a low rate of compliance with follow-up. The large burden of uncontrolled hypertension and associated CVD comorbidity among PLWH poses a substantial public health concern in SSA, and tailored interventions to mitigate the impending epidemic of preventable morbidity and mortality in this population are needed. To improve blood pressure control among PLWH in SSA, proven, multi-component interventions need to be adapted and implemented. A multifaceted community healthworker-based program holds potential for controlling blood pressure among those with HIV. There are currently no published interventions that have been shown to effectively improve blood pressure control among Tanznians with HIV in a contextually appropriate manner. Studies are needed to develop and assess such interventions.

# **Rationale:**

In the Kilimanjaro Region of Tanzania, we previously found high rates of hypertension among PLWH, but very low rates of blood pressure control. Among 500 PLWH, 35% had hypertension, 67% were unaware of their hypertension diagnosis, less than 3% were taking antihypertensive medications and 90% had uncontrolled blood pressure.^26^ Concerningly, 31% of hypertensive Tanzanians with HIV had ischemic changes on electrocardiography and greater than half had intermediate to high 10-year risk for an atherosclerotic CVD event^27,28^—indicating that these patients are on an accelerated pathway towards premature CVD-related morbidity and mortality. The large burden of uncontrolled hypertension and associated CVD comorbidity among PLWH poses a substantial public health concern in SSA, and tailored interventions to mitigate the looming epidemic of preventable morbidity and mortality in this population are needed.

This project will adapt and assess the feasibility of a community healthworker-based counseling program to Tanzania as a method in improving blood pressure control in PLWH. Prior research in Tanzania has shown that community health workers (CHWs) can deliver hypertension-related counseling to PLWH, and that such counseling is both feasible and acceptable.^29^ Therefore, there is reason to belive that an adapted COBRA program, tailored to PLWH will be acceptable to PLWH and improve blood pressure control.

# **Broad Objective:**

To adapt the COBRA program to the HIV clinic setting in Tanzania, and to evaluate the feasibility and acceptability of this program.

**Specific Objectives:**

**Specfic Objective 1:** To adapt the COBRA counseling program for improving blood pressure control among PLWH in northern Tanzania.

**Specific Objective 2:** To assess the feasibility, acceptability of the adapted COBRA-TZ program and estimate its impact on blood pressure control among PLWH in northern Tanzania.

**Methods:**

**Setting:** Moshi is located in the Kilimanjaro region of Northern Tanzania. It has a population of 184,292 people and includes both Moshi Rural and Moshi Urban Districts. It is home to Kilimanjaro Christian Medical Centre (KCMC) which is a referral hospital for northwestern Tanzania and a regional training center. KCMC and Duke University (Durham, NC, USA) have developed a research infrastructure with proven success in NIH funded grants. Many successful research projects have been conducted through KCMC with the help of an experienced team of Tanzanian research nurses and data entry technicians. This study will be conducted at the Majengo Care and Treatment Center (MCTC) and Pasua Care and Treatment Center (PCTC) in Moshi, Tanzania.

**Study Design:** This study is a mixed-methods design and will include a a qualitative component and a prospective cohort. Using the ADAPT-ITT model, the COBRA program will be adapted for PLWH in northern Tanzania, and the adapted COBRA-TZ program will be implemented by community health workers in the region. Quantitative and qualitative data on measures of feasibility and acceptability and before and after blood pressure measurements and hypertension knowledge level will be measured. Qualitative information regarding barriers to hypertension and acceptability of the COBRA program among patients and providers will be obtained using focus groups and in-depth interviews.

**Study Population:** We plan to enroll 100 PLWH from MCTC and PCTC to assess the feasibility of the COBRA-TZ program. In addition, up to 24 of these participants and up to 14 health care providers involved in the intervention will be recruited for focus group discussions and in-depth interviews to explore barriers and faciliatorators of intervention adoption and perceived intervention benefits. All participants will be 18 years of age or older and will be able to speak Swahili or English. To account for possible dropouts, we anticipate up to 150 people in total will participate in the two parts of this study. We note that a related study, which helped to inform this protocol (The Prevalence of Hypertension and Acute Coronary Syndrome in the Emergency Department, Linkage to Care, and association with HIV in Moshi, Tanzania, NIMR/HQ/R.8a/Vol.IX/2580) is approved to enroll up to 6200 people.

### **Inclusion/ Exclusion Criteria:**

For participants in the COBRA-TZ feasibility study, PLWH who are ≥18 years of age, receiving routine HIV care at MCTC and PCTC and have persistently elevated blood pressure measurements will be eligible for enrollment. Specifically, those with initial elevated blood pressure measurements (systolic blood pressure ≥ 140 mmHg or diastolic blood pressure ≥ 90 mmHg) will have blood pressure measured again at least 10 minutes later. All patients with elevated blood pressures on both measurements will be asked to return to the clinic within 1-2 weeks for repeat measurement. This is similar to the procedure in our previous study.^14^ Those with a repeat systolic blood pressures > 140 and/or diastolic blood pressures > 90 will be invited to participate in the study. The mean of these three measurements will be considered as the baseline value for a given participant.

Individuals younger than 18 years, those with systolic blood pressure less than 140 and diastolic blood pressure less than 90, or those who are not enrolled in HIV care at the study site clinics will not be eligible for the study.

**Sample Size Calculation:**

This is a pilot study with primary outcomes of feasibility and acceptability. Therefore, we will enroll 100 patients to obtain feasibility and acceptability. This pilot data will be used to design future larger studies to rigorously test the effect of the adapted intervention on clinical outcomes such as long-term blood pressure control. Qualitative work with up to 24 of these participants and up to 14 health care providers is estimated to reach 90% saturation for identifying relevant usability themes.^30^

**Instruments/Procedures:**

**Adapt the community-based hypertension counseling program (Specific Objective 1):** Using the ADAPT-ITT model,^31^ an interdisciplinary team of stakeholders will adapt the COBRA program to address barriers to hypertension care among PLWH in Tanzania. Key components of COBRA include home-based patient education, blood pressure monitoring, provider training that includes use of an algorithm for appropriate pharmacotherapy, a designated hypertension care coordinator, and subsidies for patient travel and medications.^20^

Over 8 months, intervention adaptation will occur iteratively through twice-monthly meetings between the study team and the interdisciplinary design team (DCT). Design Consultation Team members will not interact with identifiable research data. That said, some may voluntarily choose to disclose private information (such as their own experience with hypertension and or HIV) during the consultation process. We will document adaption using the following ADAPT-ITT steps: ***1-2. Assessment and Decision****:* Analysis from our previously collected data regarding barriers to and facilitators of hypertension care for PLWH in Tanzania will be used to synthesize the needs of the target population and identify opportunities for CHWs to meet these needs through the adapted intervention. The DCT will review the original COBRA material from the existing protocols and manuscripts, and through collaborative group discussions and consensus, will propose iterative adaptions. Following this input, the study team will develop an outline of the adapted intervention material and will draft the scope of work of the CHW and the intervention curriculum. ***3. Administration****:* We will invite DCT members to experience the intervention as a patient would, by interacting with the CHW in observed mock trial runs of the adapted intervention. The study team will observe the mock trial run, deliberate about modules that were successful and identify modification to enhance impact. DCT members will participate in debriefing exercises facilitated by the study team to discuss further adaptions. The study team will then draft the adapted intervention content. ***4-6. Production, Topical experts and Integration****:* The experience of the observed mock trials will be used to produce a full draft of the intervention. The study team will review the full intervention protocol with the DCT, MCTC and PCTC providers, and with the Moshi Community Advisory Board. The team will discuss input, reach consensus, and make modifications for the final intervention. ***7-8. Training and Testing***: The adapted intervention will be pre-tested with a final trial run with 5 additional purposively selected participants from the target population. Following the final trial run, the DCT will meet to obtain feedback and explore challenges and solutions. This feedback will be used to refine and finalize intervention content and processes.

**Assess feasibility and acceptability of the adapted community-based counseling program (Specific Objective 2):**

Key staff, including community health workers, MCTC and PCTC nurses, and MCTC and PCTC physicians, will be trained with the adapted COBRA-TZ. This team will determine the feasibility and acceptability of the COBRA-TZ program with 100 participants recruited from PLWH receiving care at MCTC or PCTC.

To recruit participants, a member of the research team will approach patients who are presenting to MCTC or PCTC for routine HIV care. The research team member will invite the patient to a private space to discuss the research study and assess participant interest in participation. This discussion, including discussion of inclusion criteria (including history of HIV), will occur in a completely private space. Utmost attention will be paid to maintaining strict confidentiality of participants at all points of the study, including recruitment, enrolment, and follow-up. Participants will only be identified by a unique study ID, and personal identifying information will not be entered into any study databases. Participants’ HIV status will not be disclosed to anyone outside of their normal CTC care team and the study team.

Content and structure of the adapted program is dependent upon formative work from Specific Objective 1, however, we anticipate the program to include CHW-delivered hypertension counseling and blood pressure monitoring at least every 3 months during routine follow-up appointments in the HIV clinic and referral to an HIV provider for those with uncontrolled blood pressure. At enrollment and at 6-month follow-up, we will assess: (1) blood pressure, (2) antihypertensive adherence via the Voils measure of adherence,^32^ (3) body mass index (4) waist circumference, (5) cardiovascular disease risk score,^33^ and (6) hypertension knowledge via the Hypertension Knowledge-Level Scale.^34^ A member of the research team will meet with participants to conduct these asessments and will also be readily available by phone in between the in-person assesments to actively monitor for adverse events related to the intervention.

COBRA-TZ Content: Hypertension education and counseling topics covered during visits will include: complications of uncontrolled hypertension, lifestyle modification for controlling hypertension such as weight management strategies, physical activity, smoking cessation, alcohol reduction, dietary recommendations, and additional topics based on data generated in Specific Objective 1. Each session is estimated to last 30-60 minutes. CHWs will meet with patients during their HIV clinic visits every three months, for a total of at least three visits (at 0, 3, and 6 months). CHWs will measure blood pressure at each clinic visit, and in accordance with Tanzanian guidelines,^35^ participants with severe hypertension (i.e., SBP ≥ 160mmHg and/or DBP ≥ 100mmHg) will be referred immediately for pharmacotherapy. Those with persistent hypertension (i.e., SBP > 140mmHg and/or DBP > 90mmHg at any follow up visit) will also be referred for pharmacotherapy (timing parameters around this referral will be determined in Specific Objective 1). The CHW will track all referrals, and providers will use the adapted COBRA-TZ treatment algorithm for pharmacotherapy guidance. Subsidies may be provided to overcome financial barriers for accessing antihypertensive agents, as determined during the Specific Objective 1 adaptation process. We will compare participant hypertension knowledge pre and post 6-month follow-up using the Hypertension Knowledge Level Scale survey.^34^ We will use simple descriptive statistics to assess measures of feasibility (i.e. the percentage of eligible MCTC patients who enroll, the percentage of enrolled participants who complete the 6-month follow-up, the percentage of completed community health worker sessions planned, the percentage of non-participant stakeholders who “agree” or “completely agree” to the four items of the Feasibility of Intervention Measure, and if costs are as budgeted) and fidelity (i.e. the proportion of intervention components successfully delivered as designed which will be assessed by audiorecording a portion of the counseling sessions). Acceptability will be assessed via in-depth interviews of patient, community health workers, and provider participants as well as focus group discussions with providers and health adminstrators (see below).

**In-depth Interview & Focus Group Methodology (Specific Objective 2):** Approximately twenty in-depth inteviews and approximately two focus group discussions will be conducted with patients, providers, and community-health workers to assess the acceptability of COBRA-TZ program. We will conduct exit in-depth interviews (IDIs) with a subset of participants enrolled in COBRA-TZ (n=20) and will conduct approximately 2 focus group discussions with health care providers and administrators from MCTC and PCTC (n=14). The sample of participants will be adults with HIV and hypertension who are already enrolled in the COBRA-TZ intervention and will be purposively selected by study team members to ensure diversity in gender, age, and hypertension care engagement.

The health care providers and administrators will include HIV and hypertension physicians employed at MCTC and PCTC, nurses from MCTC and PCTC, community health workers at MCTC and PCTC, and local and national health administrative leaders from MCTC, PCTC and the Ministry of Health. Health care providers and administrators will be purposively selected by study team members to ensure diversity in gender, age, and professional experience and expertise and will be approached by a member of the research team by email, phone call or in person. Participants will provide written informed consent prior to participation. These conversations will be recorded and transcribed.

**Survey Data Collection:** Data will be collected on tablets and stored in a secure database by a trained data entry technician. Surveys for all components of this study will be verbally administered to patients and families due to variable literacy rates. They will be administered by healthcare providers in the standard medical language of English or Swahili. Phone number of the patient and will be recorded in the log and de identified. No identifying information will be entered into any study database. The log will remain in a secure, locked cabinet in the research office.

**Data Analysis:** Survey results will be analyzed through analytical techniques. Continuous variables will be reported as mean and standard deviation (SD) or median and inter-quartile range (IQR).

**Feasibility Analysis:** Reach evaluates intervention participation and intervention feasibility. Using counts and proportions we will describe the proportion of eligible participants recruited and enrolled in the pilot trial and calculate the proportion of participants who 1) attend scheduled CHW visits; 2) are referred to a provider; 3) attend a referral appointment; 4) are prescribed antihypertensives; 5) report adherence to antihypertensives at 6-month follow-up, and 6) the proportion of prescribed antihypertensives that conform to the COBRA-TZ treatment algorithm.

**Fidelity Analysis:** Implementation assesses intervention fidelity. To assess CHW fidelity to COBRA-TZ content, we will audio record and review 20% of CHW-delivered counseling sessions (5-7 sessions per month) to assess session completion and evaluate the CHW’s use of core counseling components. Fidelity to the curriculum and core counseling components will be reported as counts and proportions. CWHs will complete a COBRA-TZ fidelity checklist for each session. A score of 90% on the checklist indicates adequate intervention fidelity.

**Acceptability Analysis:** We will conduct exit in-depth interviews (IDIs) with a subset of participants enrolled in COBRA-TZ and will conduct focus group discussions (FGDs) with physicians, nurses, CHWs, and administrators from MCTC and PCTC to determine acceptability of the intervention. Participants will provide feedback about their favorite and least favorite aspects of the intervention and suggestions for modification and scale-up. IDIs will further explore why some individuals chose not to participate in COBRA-TZ or not complete all COBRA-TZ sessions. The four items of the Acceptability of Intervention Measure (AIM) ^36^will be incorporated in IDIs and FGDs and will be coded using the Theoretical Framework of Acceptability (TFA), using 7 key domains of acceptability: affective attitude, burden, perceived effectiveness, ethicality, intervention coherence, opportunity costs, and self-efficacy.^37^ TFA was previously used to explore acceptability of COBRA in Asia.^22^ Participants will also be asked about their willingness to participate in COBRA-TZ in the future and to integrate COBRA-TZ into routine care in Tanzania, and we will discuss the resources, strategies and policies needed for intervention sustainability needed beyond the study period.

**In-Depth Interview Data Analysis:** After transcription, each of the focus groups and in-depth interviews will be translated and the final transcript will be discussed by the research assistants present in the focus group for content validation. Then a thematic analysis will be undertaken for each of the main questions from the focus groups/in depth interviews. All data will be analyzed with NVivo 10.0. Latent Semantic Analysis and Sentiment Analysis will be used to investigate latent concepts or positive/negative tones behind the qualitative answers. All transcripts, audiotapes and other study documents will be kept for 6 years following conclusion of the data the conclusion of the study.

**Other Analyses:** Secondary preliminary effectiveness outcomes will include changes from baseline to 6-month follow-up in: blood pressure, antihypertensive adherence using the Voils measure of adherence,^32^ body mass index, waist circumference, CVD risk score,^33^ and hypertension knowledge as measured by the Hypertension Knowledge-Level Scale.^34^ We will compare mean SBPs, mean DBPs and the proportion of participants achieving hypertension control (SBP < 140 mmHg and DBP < 90 mmHg), from before and after the pilot intervention.

**Study Timeline:** The first steps of the project included IRB submission and planning which is currently ongoing. The Duke IRB is approved and KCMC and NIMR application is in process. Project planning for the adapting the COBRA counseling program will be ongoing until January 2024. The COBRA program will be adapted for the Tanzanian context and key staff will be trained with the adapted COBRA-TZ program from from January 2024 until September 2024. The feasibility, fidelity, and acceptability of the COBRA-TZ program will be assessed from September 2024 until December 2025.

**Investigators:**

The PI is **Dr. Francis Sakita**. He is an emergency medicine physician and head of the Emergency Medicine Department at KCMC. He has a strong research interest in cardiovascular emergencies. He will supervise all study staff and all study activities. He will also participate in intervention development, budget management, data analysis, and manuscript writing.

**Dr. Blandina Mmbaga** is a co-investigator and the onsite research director at KCMC. She will ensure that the study is carried out with fidelity. As a native swahili-speaker, medical provider, and clinical researcher, she will provide critical cultural and clinical expertise. She will assist with funds management, regulatory approval, staff supervision, and manuscript writing.

**Dr. Julian Hertz** is a co-investigator. He will assist with project planning, implementation, data analysis, and manuscript preparation. He will also assist with ECG interpretation, data management, supplies, and data quality assurance/quality control.

**Dr. Preeti Manavalan** is a co-investigator. She will assist with the adaptation of the hypertension counseling program and the mixed-methods tools to assess its feasibility.

**Dr. Nathan Thielman** is a co-investigator. He will assist project planning, implementation, data analysis, and manuscript preparation.

**Ethical Considerations:**

**Capacity Development:**

This project is based on the local needs, available resources and standard practices in Moshi. We have sought out collaborators both at Duke as well as at KCMC and have adapted our project to their suggestions and experiences. We will employ and train, Tumsifu Tarimo, a Masters student in sociology at the Institute of Social Work in Dar es Salaam, who has considerable experience performing focus groups with the Duke-KCMC research collaboration. He will oversee the focus groups and in-depth interviews and his thesis work will be nested within the study. We will continue to recruit junior facility at KCMC to assist with all research activities and collaborate with providers at MCTC, PCTC, and KCMC for publications and future research projects.

**Subject Recruitment and Compensation:**

All patients at MCTC and PCTC who meet inclusion criteria will be offered enrollment in the study. The only cost to participants is the time spent answering survey questions and time spent participating in CHW-led counseling sessions. The participants will not be compensated for their time at initial enrollment visit, but they will be provided with travel subsidies (tsh 5,000) for any additional visits to the clinic for CHW-led counseling (ie, at 3 months and 6 months). Participants who participate in in-depth interviews or focus groups will compensated for travel and provided with refreshments. The original COBRA intervention included subsidies for antihypertensive medications;^17^ the exact nature and amount of medication subsidies for participants who meet criteria for antihypertensive pharmacotherapy will be determined during the adaptation process of Specific Objective 1.

**Consent Process:**

For patients we will be utilizing a written informed consent. Participants will be provided with a description of the study objectives and procedures, along with information on data safety. Agreement to participate in the survey will serve as consent to participate in the study. The participants will be enrolled by study nurses or research assistants with training in informed consent.

### **Limitations of the Study:**

While blood pressure measurement is generally highly sensitive and specific for eventual diagnosis of hypertension, a small number of patients will be included who do not ultimately have this diagnosis. The generalizability of the data is limited as this study will be conducted at only two HIV clinics in Moshi, Tanzania. Future studies will be needed in order to generalize our results to other regions in Tanzania.

**Dissemination and Publications:**

Dissemination of results will occur through academic literature in the form of international presentations and research manuscripts as well as presentations in relevant clinical settings in Moshi. We will offer to KCMC leadership to discuss our results during the weekly Clinical Case Conference in order to educate KCMC Clinicians. Similarly, presentations of study results will be given to all healthcare providers at MCTC and PCTC. Study findings will be published in peer-reviewed journals.

**Budget:**

| **Item** | **Cost in Tanzanian Shillings** |
| --- | --- |
| Research Assistant/nurses time x 2 years | 44,160,000 |
| RN data/internet time x 6 years | 552,000 |
| Community Health Workers x 1 year | 16,560,000 |
|  |  |
| Printing .13 x sheet | 956,800 |
| Automatic BP cuff x 2 | 230,000 |
| Point of care glucometers | donated |
| In-depth interview transportation/food/time | 207,000 |
| Focus Group transportation/refreshments/time | 138,000 |
| Medication/Travel subsidies | 2,300,000 |
| Design Team Food/travel subsidies | 1,150,000 |
| Training Costs (refreshments, travel reimbursements, supplies | 1,150,000 |
|  |  |
| KCMC overhead 10% total budget | 6,741,300 |
|  | 67,403,800 |
| **Total** | **74,145,100** |
|  |  |
|  |  |
|  |  |

# **Budget Justification:**

**PERSONNEL**

**Research nurse:** Two full time research nurses/research assistants will be required for all the aims of this study. They will be employed according to the research project requirements and will be active on other projects thus supplementing their salary during periods when less participant contact is needed. The research nurses will be responsible for recruiting patients, administering surveys, and conducting follow-up groups. They will also be responsible for informed consent of participants, conducting all interviews, maintaining participant files, and communicating progress to the PI. Nurses will be paid 920,000 TZS per month. Two nurses will be full-time for 2 years (44,160,000 TZS).

**Data/phone credit:** The nurses will be given 23,000 TZS per month to cover any communication costs incurred during the study (552,000 TZS).

**Community Health Workers**: We will hire two community health workers (one at MCTC and one at PCTC) to deliver the hypertension counseling program. CHWs will be paid 690,000 TZS per month x 1 year (16,560,000 TZS).

**SUPPLIES:**

**Laboratory equipment:** Two automatic blood pressure cuffs will be needed. (230,000 TZS)

**Paper and printing costs:** (7,654,400 TZS) We request funds in order to cover paper and printing costs for the project. Informed consent, surveys and any regulatory paperwork will need printing estimating 16 pages/participant x 200 participants at 3200 pages at 299 TZS for paper and printing costs. Total funds requested 956,800 TZS.

**In-depth interview transportation and food:** Each participant will be reimbursed (5,000 TSH) for transportation plus food. We anticipate performing approximately 30 in-depth interviews (207,000 TZS)

**Focus Group transportation and food:** Each participant will be reimbursed (approximately 5,000 TSH) for transportation plus food. We anticipate performing 2 focus groups with approximately 20 total participants (138,000 TZS)

**Medication/travel subsidies:** Participants in the COBRA-TZ intervention will receive approximately 23,000 TZS each to reimburse transport and subsidize medications. (2,300,000 TZS total).

**Design Team refreshments/travel:** Members of the Design team that will adapt the COBRA program across multiple meetings will receive refreshments during the meeting and reimbursement for their travel. (1,150,000 TZS total).

**Training costs:** We will hold multiple training sessions with the CHWs and the staff at MCTC and PCTC to training them in the adapted COBRA-TZ program. We will need approximately 1,150,000 TZS to cover training supplies, refreshments, and transportation for these sessions.

**KCMC overhead** According to our funders, the US National Institute of Health guidelines, we are requesting 10% of the total KCMC budget be paid to the Good Samaritan Foundation account at KCMC hospital for the privilege of being housed at KCMC and other administrative costs.

References

1. WHO | A global brief on hypertension. *WHO*. 2013. doi: /entity/cardiovascular_diseases/publications/global_brief_hypertension/en/index.html

2. Pastakia SD, Ali SM, Kamano JH, Akwanalo CO, Ndege SK, Buckwalter VL, Vedanthan R, Bloomfield GS. Screening for diabetes and hypertension in a rural low income setting in western Kenya utilizing home-based and community-based strategies. In: *Global Health*. 2013:21.

3. Patel JWS, Joseph RE, Elizabeth LT, Nathan T, Uptal D. Neighborhood clustering of non-communicable diseases: results from a community-based study in Northern Tanzania. *BMC Public Health*. 2016;16:226. doi: 10.1186/s12889-016-2912-5

4. Naanyu V, Vedanthan R, Kamano JH, Rotich JK, Lagat KK, Kiptoo P, Kofler C, Mutai KK, Bloomfield GS, Menya D, et al. Barriers Influencing Linkage to Hypertension Care in Kenya: Qualitative Analysis from the LARK Hypertension Study. *Journal of general internal medicine*. 2016;31:304-314. doi: 10.1007/s11606-015-3566-1

10.1007/s11606-015-3566-1. Epub 2016 Jan 4.

5. Shah ASV, Stelzle D, Lee KK, Beck EJ, Alam S, Clifford S, Longenecker CT, Strachan F, Bagchi S, Whiteley W, et al. Global Burden of Atherosclerotic Cardiovascular Disease in People Living With HIV. *Circulation*. 2018;138:1100-1112. doi: 10.1161/CIRCULATIONAHA.117.033369

6. Subramanian S, Tawakol A, Burdo TH, Abbara S, Wei J, Vijayakumar J, Corsini E, Abdelbaky A, Zanni MV, Hoffmann U, et al. Arterial inflammation in patients with HIV. *Jama*. 2012;308:379-386. doi: 10.1001/jama.2012.6698

7. Grinspoon S, Carr A. Cardiovascular risk and body-fat abnormalities in HIV-infected adults. *The New England journal of medicine*. 2005;352:48-62. doi: 10.1056/NEJMra041811

8. Mocroft A, Reiss P, Gasiorowski J, Ledergerber B, Kowalska J, Chiesi A, Gatell J, Rakhmanova A, Johnson M, Kirk O, et al. Serious fatal and nonfatal non-AIDS-defining illnesses in Europe. *Journal of acquired immune deficiency syndromes (1999)*. 2010;55:262-270. doi: 10.1097/QAI.0b013e3181e9be6b

9. UNAIDS. *Global AIDS Update*. Geneva: Joint United Nations Programme on HIV/AIDS; 2016.

10. Tibazarwa K, Ntyintyane L, Sliwa K, Gerntholtz T, Carrington M, Wilkinson D, Stewart S. A time bomb of cardiovascular risk factors in South Africa: results from the Heart of Soweto Study "Heart Awareness Days". *International journal of cardiology*. 2009;132:233-239. doi: 10.1016/j.ijcard.2007.11.067

11. Campbell NR, Lemogoum D. Hypertension in sub-Saharan Africa: a massive and increasing health disaster awaiting solution. *Cardiovascular journal of Africa*. 2015;26:152-154.

12. Manavalan P, Minja L, Wanda L, Hertz JT, Thielman NM, Okeke NL, Mmbaga BT, Watt MH. "It's because I think too much": Perspectives and experiences of adults with hypertension engaged in HIV care in northern Tanzania. *PLoS One*. 2020;15:e0243059. doi: 10.1371/journal.pone.0243059

13. Cappuccio FP, Miller MA. Cardiovascular disease and hypertension in sub-Saharan Africa: burden, risk and interventions. *Intern Emerg Med*. 2016;11:299-305. doi: 10.1007/s11739-016-1423-9

14. Manavalan P, Madut DB, Hertz JT, Thielman NM, Okeke NL, Mmbaga BT, Watt MH. Hypertension burden and challenges across the hypertension treatment cascade among adults enrolled in HIV care in northern Tanzania. *Journal of clinical hypertension (Greenwich, Conn)*. 2020;22:1518-1522. doi: 10.1111/jch.13929

15. Hyle EP, Mayosi BM, Middelkoop K, Mosepele M, Martey EB, Walensky RP, Bekker LG, Triant VA. The association between HIV and atherosclerotic cardiovascular disease in sub-Saharan Africa: a systematic review. *BMC public health*. 2017;17:954. doi: 10.1186/s12889-017-4940-1

16. Sliwa K, Carrington MJ, Becker A, Thienemann F, Ntsekhe M, Stewart S. Contribution of the human immunodeficiency virus/acquired immunodeficiency syndrome epidemic to de novo presentations of heart disease in the Heart of Soweto Study cohort. *European heart journal*. 2012;33:866-874. doi: 10.1093/eurheartj/ehr398

17. Jafar TH, Gandhi M, de Silva HA, Jehan I, Naheed A, Finkelstein EA, Turner EL, Morisky D, Kasturiratne A, Khan AH, et al. A Community-Based Intervention for Managing Hypertension in Rural South Asia. *The New England journal of medicine*. 2020;382:717-726. doi: 10.1056/NEJMoa1911965

18. Jafar TH, Hatcher J, Poulter N, Islam M, Hashmi S, Qadri Z, Bux R, Khan A, Jafary FH, Hameed A, et al. Community-based interventions to promote blood pressure control in a developing country: a cluster randomized trial. *Annals of internal medicine*. 2009;151:593-601. doi: 10.7326/0003-4819-151-9-200911030-00004

19. Jafar TH, Islam M, Bux R, Poulter N, Hatcher J, Chaturvedi N, Ebrahim S, Cosgrove P, Hypertension Research G. Cost-effectiveness of community-based strategies for blood pressure control in a low-income developing country: findings from a cluster-randomized, factorial-controlled trial. *Circulation*. 2011;124:1615-1625. doi: 10.1161/CIRCULATIONAHA.111.039990

20. Jafar TH, Jehan I, de Silva HA, Naheed A, Gandhi M, Assam P, Finkelstein EA, Quigley HL, Bilger M, Khan AH, et al. Multicomponent intervention versus usual care for management of hypertension in rural Bangladesh, Pakistan and Sri Lanka: study protocol for a cluster randomized controlled trial. *Trials*. 2017;18:272. doi: 10.1186/s13063-017-2018-0

21. Jafar TH, Samad Z, Bloomfield GS. Parallel community solutions for cardiovascular risk reduction. *Lancet (London, England)*. 2019;394:1207-1208. doi: 10.1016/S0140-6736(19)31995-6

22. Jafar TH, Silva A, Naheed A, Jehan I, Liang F, Assam PN, Legido-Quigley H, Finkelstein EA, Ebrahim S, Wickremasinghe R, et al. Control of blood pressure and risk attenuation: a public health intervention in rural Bangladesh, Pakistan, and Sri Lanka: feasibility trial results. *Journal of hypertension*. 2016;34:1872-1881. doi: 10.1097/HJH.0000000000001014

23. Whitworth JA. 2003 World Health Organization (WHO)/International Society of Hypertension (ISH) statement on management of hypertension. *Journal of hypertension*. 2003;21:1983-1992. doi: 10.1097/01.hjh.0000084751.37215.d2

24. Hassan HC, Howlin K, Jefferys A, Spicer ST, Aravindan AN, Suryanarayanan G, Hall BM, Cleland BD, Wong JK, Suranyi MG, et al. High-sensitivity troponin as a predictor of cardiac events and mortality in the stable dialysis population. *Clinical chemistry*. 2014;60:389-398. doi: 10.1373/clinchem.2013.207142

25. Perman G, Rossi E, Waisman GD, Aguero C, Gonzalez CD, Pallordet CL, Figar S, Gonzalez Bernaldo de Quiros F, Canning J, Soriano ER. Cost-effectiveness of a hypertension management programme in an elderly population: a Markov model. *Cost effectiveness and resource allocation : C/E*. 2011;9:4. doi: 10.1186/1478-7547-9-4

26. Hertz JT, Prattipati S, Kweka GL, Mlangi JJ, Tarimo TG, Mmbaga BT, Thielman NM, Sakita FM, Rubach MP, Bloomfield GS, et al. Prevalence and predictors of uncontrolled hypertension, diabetes, and obesity among adults with HIV in northern Tanzania. *Glob Public Health*. 2022:1-13. doi: 10.1080/17441692.2022.2049344

27. Manavalan P, Madut DB, Hertz JT, Thielman NM, Okeke NL, Mmbaga BT, Watt MH. Hypertension among adults enrolled in HIV care in northern Tanzania: comorbidities, cardiovascular risk, and knowledge, attitudes and practices. *Pan Afr Med J*. 2022;41:285. doi: 10.11604/pamj.2022.41.285.26952

28. Prattipati S, Sakita FM, Tarimo TG, Kweka GL, Mlangi JJ, Maro AV, Coaxum LA, Galson SW, Limkakeng AT, Rugakingira A, et al. Prevalence and Correlates of Ischemic ECG Findings among Adults With and Without HIV in Tanzania. *Global heart*. 2022;17:38. doi: 10.5334/gh.1127

29. Manavalan P, Madut DB, Wanda L, Msasu A, Mmbaga BT, Thielman NM, Watt MH. A community health worker delivered intervention to address hypertension among adults engaged in HIV care in northern Tanzania: Outcomes from a pilot feasibility study. *Journal of clinical hypertension (Greenwich, Conn)*. 2022;24:1095-1104. doi: 10.1111/jch.14518

30. Nielsen J, Landaeuer T. A Mathematical Model of the Finding of Usability Problems. Paper/Poster presented at: Proceedings of the SIGCHI Conference on Human Factors in Computing Systems - INTERCHI ’93; 1993, 1993;

31. Wingood GM, DiClemente RJ. The ADAPT-ITT model: a novel method of adapting evidence-based HIV Interventions. *Journal of acquired immune deficiency syndromes (1999)*. 2008;47 Suppl 1:S40-46. doi: 10.1097/QAI.0b013e3181605df1

32. Voils CI, Maciejewski ML, Hoyle RH, Reeve BB, Gallagher P, Bryson CL, Yancy WS, Jr. Initial validation of a self-report measure of the extent of and reasons for medication nonadherence. *Med Care*. 2012;50:1013-1019. doi: 10.1097/MLR.0b013e318269e121

33. Gaziano TA, Young CR, Fitzmaurice G, Atwood S, Gaziano JM. Laboratory-based versus non-laboratory-based method for assessment of cardiovascular disease risk: the NHANES I Follow-up Study cohort. *Lancet (London, England)*. 2008;371:923-931. doi: 10.1016/S0140-6736(08)60418-3

34. Erkoc SB, Isikli B, Metintas S, Kalyoncu C. Hypertension Knowledge-Level Scale (HK-LS): a study on development, validity and reliability. *International journal of environmental research and public health*. 2012;9:1018-1029. doi: 10.3390/ijerph9031018

35. *Standard treatment guidelines & national essential medicines list: Tanzania mainland*. 5th ed. Dar es Salaam: Ministry of Health, Community Development, Gender, Elderly and Children; 2018.

36. Weiner BJ, Lewis CC, Stanick C, Powell BJ, Dorsey CN, Clary AS, Boynton MH, Halko H. Psychometric assessment of three newly developed implementation outcome measures. *Implementation science : IS*. 2017;12:108. doi: 10.1186/s13012-017-0635-3

37. Sekhon M, Cartwright M, Francis JJ. Acceptability of healthcare interventions: an overview of reviews and development of a theoretical framework. *BMC health services research*. 2017;17:88. doi: 10.1186/s12913-017-2031-8
